# The Investigation of a SAW Oxygen Gas Sensor Operated at Room Temperature, Based on Nanostructured Zn*_x_*Fe*_y_*O Films

**DOI:** 10.3390/s19133025

**Published:** 2019-07-09

**Authors:** Lin Shu, Tao Jiang, Yudong Xia, Xuemin Wang, Dawei Yan, Weidong Wu

**Affiliations:** 1 Science and Technology on Plasma Physics Laboratory, Research Center of Laser Fusion, China Academy of Engineering Physics, Mianyang 621900, Sichuan, China; 2 School of Physical Science and Technology, Southwest Jiaotong University, Key Laboratory of Advanced Technology of Materials, (Ministry of Education), Chengdu 610031, China; 3 Collaborative Innovation Center of IFSA (CICIFSA), Shanghai Jiao Tong University, Shanghai 200240, China

**Keywords:** Zn*_x_*Fe*_y_*O nanostructures, oxygen gas sensor, room temperature, surface acoustic wave

## Abstract

In this paper, we report a wireless gas sensor based on surface acoustic waves (SAW). For room temperature detection of oxygen gas, a novel nanostructured Zn*_x_*Fe*_y_*O gas-sensitive film was deposited on the surface of a SAW resonator by an oblique magnetron co-sputtering method. The measurements of X-ray diffraction (XRD) and a scanning electron microscope (SEM) showed that the crystal phase composition and the microstructures of Zn*_x_*Fe*_y_*O films were significantly affected by the content of Fe. The experimental results showed that the sensors had a good response to O_2_ at room temperature. The max frequency shift of the sensors reached 258 kHz as the O_2_ partial pressure was 20%. Moreover, X-ray photoelectron spectroscopy (XPS) was performed to analyze the role of Fe in the sensitization process of the Zn*_x_*Fe*_y_*O film. In addition, the internal relationship between the Fe content of the film and the sensitivity of the sensor was presented and discussed. The research indicates that the nanostructured Zn*_x_*Fe*_y_*O film has a good potential for room temperature O_2_ gas detection applications.

## 1. Introduction

In recent years, the demand for oxygen gas detection is growing in a wide range of applications, including environment protection, food security, automotive, and engineering fields [1,2,3]. Among numerous oxygen gas sensors, sensors based on surface acoustic wave (SAW) techniques have attracted lots of attentions for their advantages of high sensitivity, fast response, high accuracy and capacities of wireless and passive detection [4]. Generally, a SAW sensor is made up of a SAW oscillator and a gas sensitive film. The SAWs excited by the resonator propagate near the substrate surface and are very sensitive to environmental changes near the substrate surface. Owing to absorbing target gas molecules, the mass density and electrical quantity of the sensitive film changes, leading to a variation in the propagation characteristics of SAWs, such as propagation velocity, phase, propagation mode, insertion loss, etc. Obviously, the performance of a gas-sensitive film largely determines the performance of the surface acoustic wave gas sensor. Zinc oxide (ZnO), one of the metal oxide semiconductor (MOS) materials, receives considerable attention in gas sensing applications due to its high mobility of electrons and high chemical stability [5]. 

It is known that oxygen vacancies (V_o_) play an important role in the gas sensitive behavior of MOS materials [6]. To ZnO, its zinc interstitials (Zn_i_), acting as shallow donor defects, would compete with V_o_ to absorb oxygen. Donor defects play a leading role in the chemisorption and ionization of oxygen on the surface of ZnO during gas sensing process and determine the adsorption capacity of the material [7]. However, the sensing performance of ZnO film is greatly limited at room temperature due to its large bandgap (3.37 eV). Most ZnO based gas sensors need to operate at high temperatures to generate more electron-hole pairs to enhance their gas sensing performance [8]. Nanostructured modification and doping with other metal elements have proven to be the most effective means to improve the gas sensing performance of ZnO at room temperature. The nanostructured ZnO provides a large specific surface area to absorb the target gas, while the doped metal molecules in ZnO act as electron donors and provide excess carriers to conductance band [9]. Both of these two factors will significantly improve the sensor response. For example, Marcu and Viespe developed a room temperature hydrogen and deuterium sensor based on a SAW delay-line and ZnO nanowires. They focused on the effects of the morphology of ZnO nanowires on the response to target gas [10]. Motaung et al. enhanced the response of ZnO film to CO_2_ by doping it with Mn, and studied the correlation between the amount of dopant and gas sensing properties of the Mn-doped ZnO film [11]. Hardan et al. successfully reduced the operation temperature of an acetone gas sensor by doping Cr in ZnO [12].

Among these dopants, the doped Fe ions introduced ferric interstitials in the sensitive films, which act as shallow donor defects, and competed with Vo and Zni in the adsorption of oxygen, resulting in a significantly improvement of gas sensing performance of the sensor [13]. Moreover, the doping of Fe element could obviously change the morphology of the sensitive film, leading to a formation of nanostructure [14]. The nanostructured sensitive film has a larger specific surface area and more structural defects, which also enhances the gas detection capacity of the sensor.

At present, there are many methods to prepare Zn*_x_*Fe*_y_*O gas-sensitive materials, such as sol-gel, hydrothermal, combustion, and sputtering et al. [15,16,17]. Among these methods, physical sputtering deposition technology has the characteristics of good controllability, high repeatability, and good consistency. However, due to the high energy of sputtered particles in the process of physical sputtering, they randomly distribute on the substrate surface and agglomerate to form larger grains after reaching the substrate surface. Therefore, compared with other methods to prepare Zn*_x_*Fe*_y_*O gas-sensitive films, the films prepared by physical sputtering have smaller specific surface area and surface defect density, which is not conducive to the application of gas sensing [18]. Herein, an oblique magnetron co-sputtering method was used to synthesize Zn*_x_*Fe*_y_*O films on SAW chips. During the deposition process, the substrate rotates uniformly to obtain nanostructured Zn*_x_*Fe*_y_*O. The response of SAW sensors coated with Zn*_x_*Fe*_y_*O sensing films to oxygen gas was examined at room temperature. It is expected that the concentration of Fe in Zn*_x_*Fe*_y_*O film would significantly affect the film surface morphology, as well as the sensing performance of the sensors. Therefore, the dependence of the sensor response to oxygen gas on different Zn*_x_*Fe*_y_*O films was studied and discussed.

## 2. Experimental Setup

**Figure 1:**
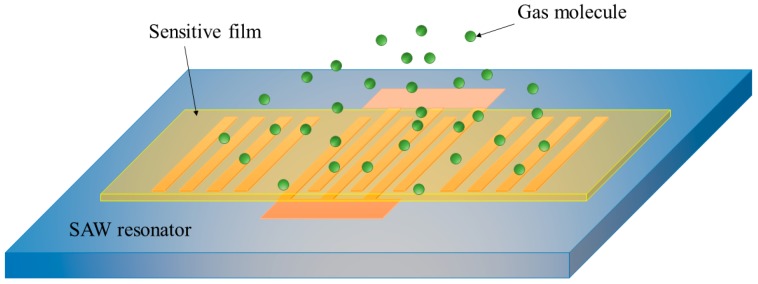
Structural diagram of SAW gas sensor based on a one-port resonator.

In this work, a one-port SAW resonator-based gas sensor was simulated and designed by the finite element method (FEM) in COMSOL. The details of the SAW resonator are presented in Table 1. It should be noted that the center resonance frequency of the sensor was designed to be 335 MHz to meet the requirements of ISM (Industrial Scientific Medical) band for wireless applications in the future. Moreover, the quality factor (*Q*-factor) of the sensor was optimized to be >6000 in simulation. Since the Q factor of SAW sensor is higher, its sensitivity will be higher [19]. In the experiments, the SAW resonator was fabricated by photolithography and lift-off technics. The structural diagram of the realized SAW sensor is presented in Figure 1.

**Figure 2:**
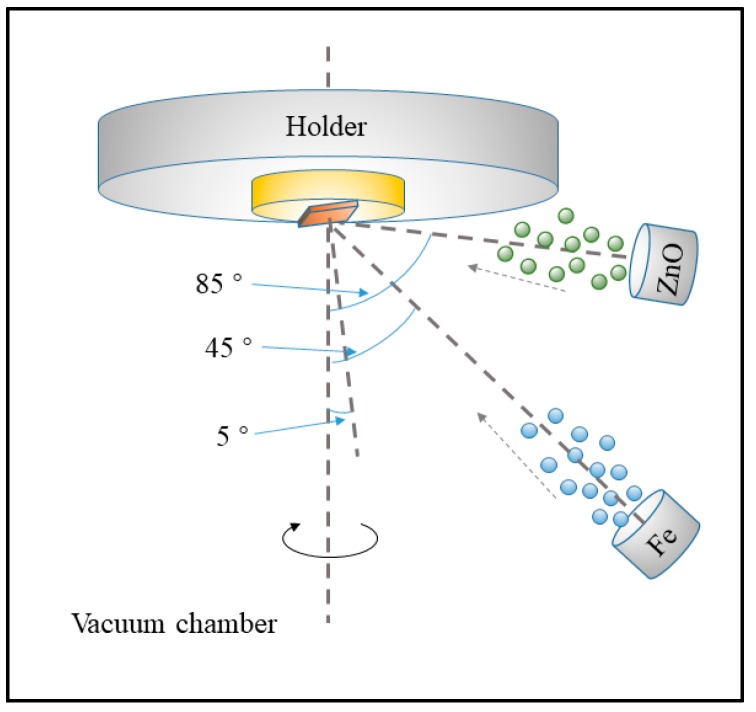
The schematic diagram of co-sputtering oblique angle deposition system for preparing of ZnxFeyO film.

The Zn*_x_*Fe*_y_*O sensitive films, covered on the top of SAW chips, was synthesized by a co-sputtering oblique angle deposition method. The two materials, including ZnO (99.9%) and Fe (99.95%), were installed in the sputtering targets with different sputtering oblique angles, respectively, as shown in Figure 2. Here, we used RF power for ZnO sputtering and DC power for Fe sputtering. Taking the normal line of the substrate holder as the reference line, the angle between the normal lines of the ZnO target and the reference line were 85°, while the angle of Fe target was 45°. Notably, the SAW substrates were also obliquely mounted on the substrate holder and the angle between its normal line and reference line was 5°. Meanwhile, the substrate spun at a constant speed in the whole sputtering process. The distance between the substrate and the ZnO and Fe targets were 9 and 13 cm, respectively. Experimentally, the sputtering current of the Fe target varied from 0.02 to 0.1 A, while the sputtering power of ZnO target was settled to 100 W. The content of Fe in the Zn*_x_*Fe*_y_*O film was adjusted by changing the sputtering power of the Fe target. Before measurement, the SAW chips were annealed at 500 °C for 2 h in the air and cooled down naturally to room temperature.

The crystal structures and surface morphology of the ZnO sensitive films were characterized by X-ray diffraction (XRD, Cu-Kα, Bede-D1, Bede Co., Sunderland, UK) and SEM (JSM-6460, JEOL Ltd., Tokyo, Japan). Meanwhile, the elemental composition of the prepared films was determined by energy dispersive X-ray spectroscopy (EDX) and an X-ray photoelectron spectroscope (XPS).

**Figure 3:**
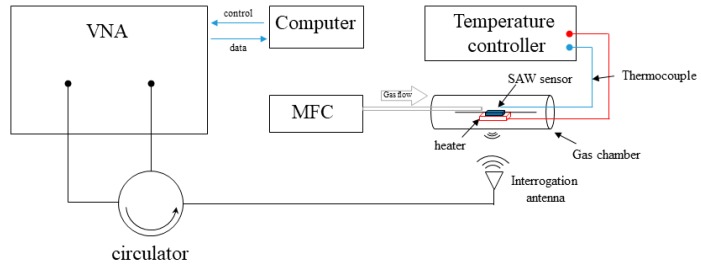
The schematic diagram of the experimental setup.

Herein, the SAW sensors were attached to a *λ*/4 dipole antenna, which was made of 0.5 mm diameter copper wire, for wireless detection. The schematic diagram of the experimental setup is presented in Figure 3. It mainly contained a mass flow controller (MFC) unit (E-lite, Beijing, China), a vector network analyzer (VNA, Agilent E5071b, Agilent Technologies Inc., Santa Clara, CA, USA), and a temperature-controlled gas cell. The realized SAW gas sensors were continuously measured, with O_2_ gas partial pressure (PO_2_) increasing from 0.1% to 20%. The operating temperature was 25 °C. The measurements of the reflection-scattering parameters (S_11_) were acquired and processed in a computer by homemade software. Notably, we used two methods to evaluate the gas sensitivity of the sensors. One was the ‘breathing’ measurement, in which target gas (O_2_) and background gas (N_2_) was filled into the chamber successively to enable the sensors to reach full adsorption and desorption of target gas molecules. In this test, we can study the sensitivity and response time of the sensors. The other was the ‘stepping’ measurement, in which the PO_2_ exposed to the sensors increased from 0% to 20% by step, and then decreased to 0%. Here, we focus on the repeatability and hysteresis of the sensors in the gas sensing applications.

## 3. Results and Discussions

### 3.1. Characterization of Zn_x_Fe_y_O Sensitive Films

In the experiments, there were five samples with different Zn*_x_*Fe*_y_*O films prepared. The sputtering of Fe target was 0, 0.02, 0.05, 0.08, and 0.1A, respectively. The composition of the films was characterized by EDX and is presented in detail in Table 2. It is clear that the concentration of the Fe element is greatly influenced by the sputtering current of the target. 

**Figure 4:**
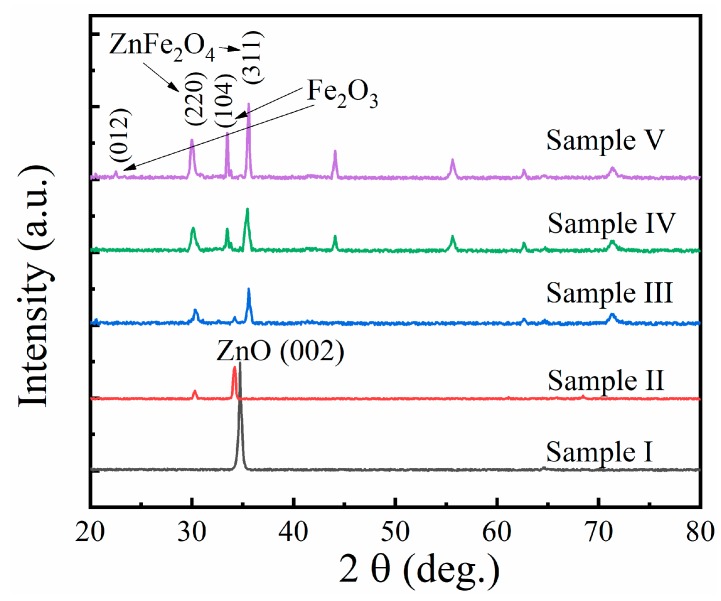
XRD patterns of Samples I–V, corresponding to the Fe concentration of 0, 3.5, 59, 70.1, and 75.5 at%, respectively.

Figure 4 shows the XRD patterns of the Zn*_x_*Fe*_y_*O samples. As the sputtering current of the Fe target was 0 A, Sample I was pure ZnO film without any doping. In Sample I, a strong diffraction peak at 34.73° can be observed, which corresponds to the (002) plane of ZnO. When the sputtering current of Fe target increased, i.e., the Fe element was introduced into ZnO film, we found that the crystal structure of the film changed significantly. When the current increased to 0.02 A, the ZnO (002) diffraction peak had a blue shift, which was defined as the negative shifts of the crystal planes, indicating the increase of cell parameters of the film structure. At the same time, the intensity of the diffraction peak was also significantly reduced. These indicate that the introduction of a small amount of the Fe element will reduce the c-axis orientation of thin film, which led to a change in the crystalline structure of the film. In addition, a weak diffraction peak of Sample II appeared at 2θ = 30.3°, corresponding to the (220) lattice plane of ZnFe_2_O_4_, which indicates that the ZnFe_2_O_4_ will be synthesized in preference to Fe_2_O_3_ in the co-sputtering deposition process. With a further increase of sputtering current of the Fe target to 0.05 A, the ZnO (002) diffraction peak of Sample III became weak, indicating that the crystal texture of the films was rebuilt by introduction of Fe. The main diffraction peaks of Sample III, existing at 30.36° and 35.57°, were coincident with the characteristic peak of ZnFe_2_O_4_ (PDF #73-1963). According to the results obtained by EDX, the content ratio of Fe and Zn in Sample III was 59 at%, 41 at%, respectively. As no other characteristic diffraction peaks of the sample could be observed, we considered that the crystal structure in the film is only composed of ZnO and ZnFe_2_O_4_. The Fe sputtering current of Sample IV was 0.08 A and the proportion of Fe to Zn in the sample was 70.1 at%:29.1 at%, which exceeded 2:1. The diffraction peak of Fe_2_O_3_ (104) planes was observed in the XRD pattern. At the same time, we find that the characteristic peak intensity of ZnFe_2_O_4_ increases, showing an improvement in crystal texture and orientation of ZnFe_2_O_4_. Sample V was obtained by enhancing the Fe sputtering current to 0.1 A. It could be seen from the XRD patterns that not only was the intensity of Fe_2_O_3_ (104) peak enhanced, but the Fe_2_O_3_ (012) peak also appeared in the sample. It has been reported that the exposure to these two crystal surfaces is beneficial to improve the sensing characteristics of Fe_2_O_3_ materials [20]. Moreover, the phenomenon of blue shift also existed in the ZnFe_2_O_4_ (220) crystal plane. Compared with Sample III, the shifts in peak position Δ(2θ) were 0.24° and 0.35° for Sample IV and Sample V, respectively. This reveals the change in lattice parameters and cell volumes of ZnFe_2_O_4_ with the increased Fe element content.

**Figure 5:**
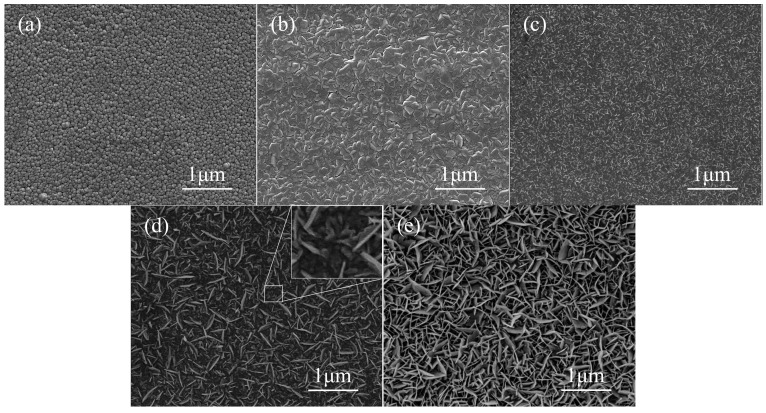
SEM images of the samples with different concentrations of Fe, (**a**) Sample I, (**b**) Sample II, (**c**) Sample III, (**d**) Sample IV, (**e**) Sample V.

Figure 5a–e show the SEM images of the samples I–V. Obviously, the surface morphology of the Zn*_x_*Fe*_y_*O films was significantly affected by the Fe content. The undoped ZnO film presented in Figure 5a was composed of ZnO crystalline columns, which were highly c-axis oriented and had good consistency, resulting in the dense surface morphology of the film. It can be seen from Figure 5b–e that the introduction of Fe had a significant impact on the surface morphology of the gas-sensitive films. With the increase of Fe content in Zn*_x_*Fe*_y_*O film, the surface of the film was gradually nano-structured. Only a small amount of nanosheets could be found on the surface of the film in Sample III. These nanosheets are evenly distributed on the surface, and no obvious agglomeration structure is found. While, in Sample IV, it can be found in Figure 5d that the amount of nanosheets increases dramatically and its size also increases significantly. However, some particles, which had not formed the nanosheets yet in their gaps, were observed, as shown in the inset of Figure 5d. With an increase of the sputtering current to 0.1 A, the size of acquired nanosheets further increased. The surface of the film was fully covered with nanosheets, as seen in Figure 5e, while the particles disappeared. This means that the film might have a larger specific surface area than the other samples, which would enhance the gas sensing performance of the sensor.

### 3.2. Characterization of SAW Resonators

**Figure 6:**
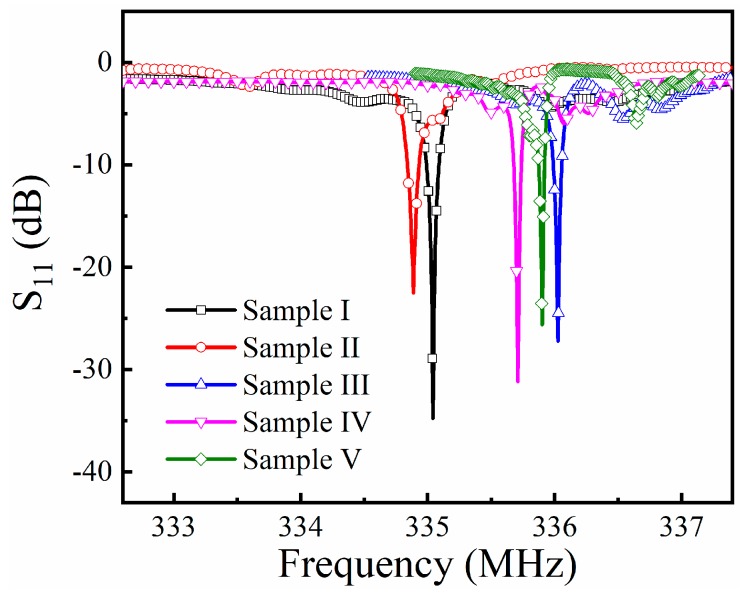
The frequency response of the sensors, measured at 25 °C.

Figure 6 shows the S_11_ measurements of the samples. The resonance frequency was 335.04 MHz, 334.89 MHz, 336.03 MHz, 335.71 MHz, and 335.90 MHz, respectively, corresponding to Samples I–V. The quality factors of the sensors were calculated by the approximate formula as presented in Equation (1),

(1) $$Q=f_{r}∕BW,$$

where *Q* stands for the quality factor, *f_r_* is the resonance frequency of the devices, and *BW* is the band width of the resonator, i.e., the full width at half maximum (FWHM) of the resonance peak. The calculated *Q*-factor of the sensors was 11,553, 7050, 10,500, 11,190, and 9595, respectively, corresponding to Samples I–V. It can be seen that the introduction of Fe to the gas-sensitive thin film will also change the center frequency of the SAW resonator and affect the *Q* factor of the device. This is because the gas-sensitive film coats on the surface of SAW resonator would take the negative effects on the periodic structure of the resonator. In general, the poorer the uniformity of the gas-sensitive film, the greater the impact on the device [21]. Hence, as the gas-sensitive film coated on Sample I was pure ZnO without doping, the film had the best consistency, leading to the strongest resonance performance among the sensors. The electrical performance of the sensors met the requirements and indicated that they have good capacity in sensing applications.

### 3.3. Gas Sensing Performance of the Sensors

**Figure 7:**
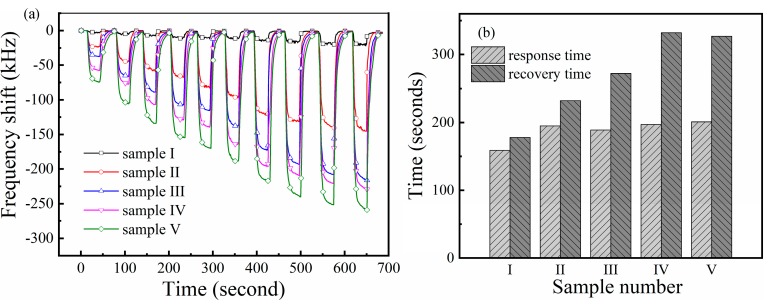
(**a**) Response of the sensors to oxygen gas with PO_2_ of 0.1%, 0.25%, 0.5 %, 0.75%, 1%, 2%, 5%, 10%, 15% and 20%, respectively; (**b**) the response and recovery time of the sensors at 25 °C.

Since the resonance frequency of each sensor is not same, we use the shift of resonant frequency as a reference to evaluate the performance of the sensor. Figure 7a presents the dynamic response of the SAW sensors to different PO_2_ in ‘breathing’ measurements. As expected, the exposure of the sensors to O_2_ firstly resulted in a rapid decrease in frequency of the sensors. Then the sensors reached the saturation response in few seconds and remained stable. When N_2_ gas blew in, the frequency of the sensors returned to the baseline soon. The samples with different films had different responses to O_2_ gas. Obviously, Sample V showed strongest response to O_2_ gas at the same PO_2_ among the samples. Its frequency shift reached −258.85 kHz as PO_2_ was 20%, which was 22 times stronger than that of Sample I. In brief, the enhancement of the sensor in gas sensing performance could be explained in terms of the large specific surface area and abundant structural defects of the doped sensitive films. This is discussed in detail in the next section. 

The response and recovery time is defined as the time the sensors spend to reach their 90% maximum response or the initial baseline. The calculated average response and recovery time of the sensors are presented in Figure 7b. The average response time of the samples was 159, 195, 189, 197, and 201 s, respectively. It was found that the sensors’ response time increases about 30% by the introduction of the Fe element, while the changes of Fe content in the film did not significantly affect the response time. It seems that the sensitive films with a stronger gas adsorption capacity need more time for desorption. 

**Figure 8:**
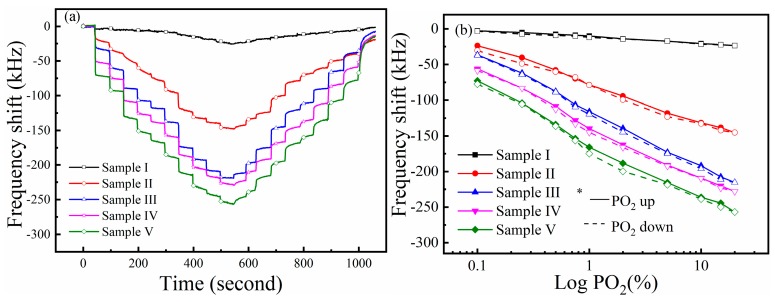
(**a**) Response of the sensors to oxygen gas in ‘stepping’ measurement; (**b**) the response and recovery time of the sensors at 25 °C.

Figure 8a presents the dynamic response of the SAW sensors in ‘stepping’ measurements. The PO_2_ firstly increased from 0% to 20%, and then decreased to 0%. Similar to previous results, the sensors had a strong response to O_2_ gas in the range from 0% to 20%. Especially for the Zn*_x_*Fe*_y_*O samples, the frequency change caused by the increase of PO_2_ was more obvious, indicating that these sensors had a good resolution in O_2_ detection. However, we also find that when the PO_2_ gets back to 0%, the response of the sensors does not return to their initial state (at least within the similar time scale as the absorption response), indicating that the adsorption of some target gas was irreversible by the films under certain conditions. This is because some of the gas molecules (residual O_2_ molecules) might be cured by chemical reactions with the sensitive film or may be trapped in the crystal cells of the material and difficult to escape, which leads to a negative effect on the repeatability of the sensors. In the follow-up study, we find that the repeatability of the sensors could be improved by increasing their working time. We speculate that this is because the number of residual O_2_ molecules on the sensitive film gradually reaches saturation after a long-term operation of the sensor. Therefore, the amount of residual O_2_ molecules on the sensitive film could no longer increase. Lastly, the sensors can be fully recovered to the initial state by heating or illuminating to the sensor. 

Figure 8b shows the relation curves between the response of the sensors and the oxygen partial pressure. The response of the sensor can be written as follows [22]:

(2) $$\Delta f_{r}\propto {\left(P_{O_{2}}\right)}^{1/m},$$

where *m* is the sensitivity coefficient. Here, we use logarithmic coordinates in the analysis. It can be found that the response of the sensors was linear to the logarithm of PO_2_. Meanwhile, the hysteresis in gas detection was observed in the measurement. That is, the sensor response differs in the PO_2_ increase process and the PO_2_ decrease process. The hysteresis error *δ_fm_*, which is the maximum deviation in output at any PO_2_ within the sensor’s specific range, is defined as follows:

(3) $$\delta_{f_{m}}=\frac{\Delta f_{m}}{Y_{\mathrm{FS}}}\times 100\%,$$

where Δ*f_m_* is the maximum deviation of the response curve in the PO_2_ increase and PO_2_ decrease processes, and *Y_FS_* is the full measurement range of the sensor in measurement. The hysteresis error of the sensors was 4.6%, 5.4%, 5.1%, 5.8%, and 5.7%, respectively, corresponding to Sample I–V. We speculate that the *δ_fm_* of the sensors is probably related to the sensor’s different rates in adsorption and desorption. Although the experimental phenomenon observed in this work was not enough to clarify the intrinsic mechanism in hysteresis, as well as the relationship between hysteresis error and response time, we can still reduce the hysteresis error by increasing the reaction time between the sensor and the target gas.

**Figure 9:**
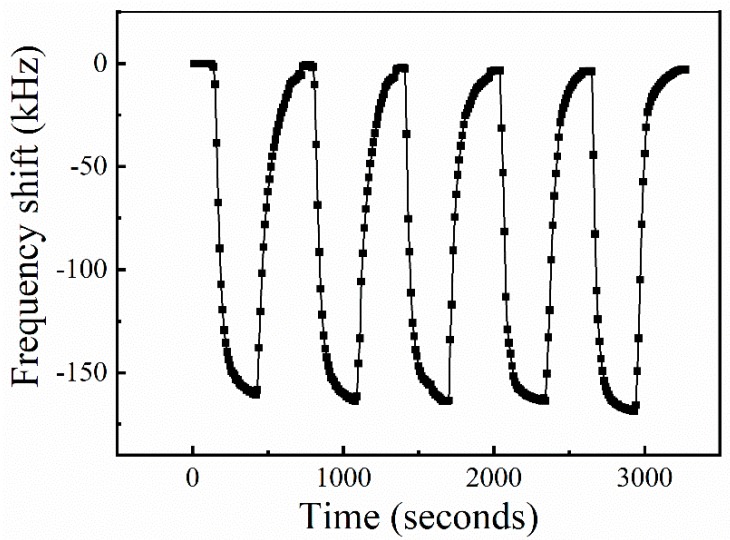
The repeatability measurement of the sensor (Sample V) with O_2_ concentration of 1% at room temperature.

The long-term repeatability of the sensor (Sample V) is verified by switching the gas from pure N2 to 1% O_2_, alternately. It should be pointed out that the sensor continuously operated in an oxygen-rich environment for over 200 h at this time. The curve of frequency shift vs. time is presented in Figure 9. It can be found that the sensor has almost the same frequency shift when exposed to 1% O_2_ at each cycle. The standard deviation of the response is 3.96. Moreover, compared to previous results (Sample V exposed to 1% O_2_), the calculated deviation error is within 2.5%, indicating good long-term repeatability of the sensors.

### 3.4. Sensing Mechanism of Zn_x_Fe_y_O Sensitive Films on O_2_ Gas

**Figure 10:**
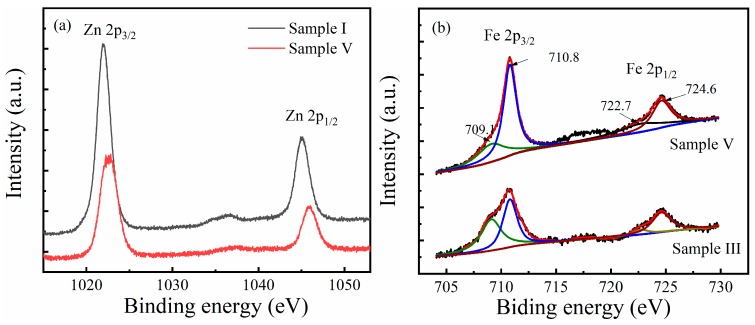
(**a**) The Zn 2p XPS spectra for Sample I and Sample V. (**b**) The Gaussian fittings in Fe 2p XPS spectra for Sample III and Sample V, showing relative weights of Fe^2+^ and Fe^3+^.

From the experimental results, the improvement of the sensors in gas detection can be attributed to the nanostructure introduced by the Fe dopant. With the increase of the Fe content, the amount of nanosheets in the sensitive film increased, which significantly increased the specific surface area of the sensitive film, enabling it to absorb more gases, thus improving the detection sensitivity of the sensor. However, Sample II, which had no obvious nano-structured feature on its surface, significantly improved in gas detection compared with Sample I. Therefore, XPS was performed to analyze the role of Fe in the sensitization process of the Zn*_x_*Fe*_y_*O film. Figure 10 shows the XPS spectra of Zn2p and Fe2p for the selected samples. From Figure 10a, the binding energy of Zn2p peaks for Sample I (undoped ZnO) were 1022.1 eV and 1045.1 eV, respectively, corresponding to the spin orbitals of Zn 2p3/2 and Zn 2p1/2. The peak positions of Zn 2p3/2 and Zn 2p1/2 in all the samples closely matched with the standard values of ZnO [23], indicating that the Zn atoms in the sample only retained the divalent oxidation state. However, the binding energy of Zn2p for Sample V increased to 1022.8 eV (for Zn2p 3/2) and 1045.8 eV (for Zn2p 1/2), respectively. The change in binding energy reflected the electronic interaction between the zinc ions and Fe ions in the Zn*_x_*Fe*_y_*O materials, which might promote the formation of free electrons and holes, thus enhancing the gas response of the sensitive films. Figure 10b shows the Fe 2p XPS spectra for Sample III and Sample V. The main peaks observed at 710.5 eV and 723.9 eV corresponded to the binding energy of Fe 2p3/2 and 2p1/2, respectively. No additional peaks corresponding to metal clusters or particles were found in any spectra. Gaussian Lorentzian curves were used to fit the Fe 2p spectra. The peak positions corresponding to the Fe^2+^ state occur at 709.1 eV and 722.7 eV, respectively, while the Fe^3+^ state occurs at 710.8 eV and 724.6 eV, respectively. Comparing the two curves, the intensities of the peaks showed an overall growth with Fe content. Meanwhile, the intensities of Gaussians corresponding to Fe^2+^ and Fe^3+^ states show that the Fe^3+^ became dominant in the samples. Moreover, the proportion of the Fe^3+^ state increased by increasing the Fe content in the Zn*_x_*Fe*_y_*O films, suggesting that the Fe^2+^ took precedence over Fe^3+^ in the deposition of Zn*_x_*Fe*_y_*O films, which was similar to the reported results in Reference [24]. 

In Zn*_x_*Fe*_y_*O films, Zn^2+^ ions are easily substituted by the Fe^3+^ ions. In the substitution process, the positive valence charge produced in the formation of Fe_Zn_∙ (Fe^3+^ substitutes Zn^2+^) is compensated by releasing electrons to maintain electrical neutrality. Then, the electrons are probably transferred to the conduction band of Zn*_x_*Fe*_y_*O materials, increasing the free carrier concentration and resulting in an improvement in the gas sensing response of the films. The reaction process can be written in equation as follows:

(4) $$\mathrm{Fe}\overset{ZnO}{\to }Fe_{Zn}^{.}+e^{-}.$$

Or

(5) $$Fe_{2}O_{3}\overset{ZnO}{\to }2Fe_{Zn}^{.}+3O_{O}^{X}+2e^{-},$$

where in the Kroger–Vink notation, Fe_Zn_^•^, is the Fe ion sitting on a zinc lattice site with one positive charge and $O_{O}^{X}$ is an oxygen ion sitting on an oxygen lattice site with a neutral charge [25]. When the sensor is exposed to O_2_ gas, oxygen molecules can easily capture the free electrons in the Zn*_x_*Fe*_y_*O film and be adsorbed on the film, causing changes in the surface conductivity and quality of the film, thus changing the resonance characteristics of the SAW sensors.

## 4. Conclusions

Zn*_x_*Fe*_y_*O nanosheets, synthesized by an oblique magnetron co-sputtering method were deposited on the surface of SAW resonators. The maximum response of the sensor (Sample V) to 20% partial pressure of O_2_ reached −258.85 kHz, which is 22 times stronger than the sensors composed of pure ZnO sensitive film. Obviously, the sensing properties of the sensors to O_2_ gas are remarkably improved by increasing the Fe content in Zn*_x_*Fe*_y_*O films. On one hand, by increasing Fe content in the film, both the size and amount of the nanosheets increase, which leads to an enhancement of the specific surface area of the film and the amount of structural defects of the film. This would significantly improve the gas adsorption capacity of the film. On the other hand, the proportion of Fe^3+^ ions increased with the increase of Fe content, resulting in an increase of Fe_Zn_∙ and free electrons, which would improve the response of the film to the target gas. However, the gas sensing hysteresis error of the sensors is over 5%. We speculate the hysteresis is probably caused by the different rates of the sensor in the adsorption and desorption of O_2_ gas. This could be reduced by heating, lighting, and increasing the reaction time of the sensors with target gas. The experimental results show that the nanostructured Zn*_x_*Fe*_y_*O, which is prepared by an oblique magnetron co-sputtering method, has a good prospect in the application of O_2_ gas detection at room temperature.

## Figures and Tables

**Table 1:** The detailed parameter of the prepared SAW resonators.

Parameters	Value
Acoustic wavelength (μm)	1.5–3.0
Strip width (μm)	2
Aperture (μm)	800
Strip numbers in IDT	101
Strip numbers in reflectors	400
Thickness of electrodes	100 nm/10 nm (Au/Ti)
Substrate material	LGS (La_3_Ga_5_SiO_14_)

**Table 2:** The composition of Zn*_x_*Fe*_y_*O films obtained by EDX.

Sample Number	Zn			Fe		
	Sputtering Method	Power (W)	Composition (at%)	Sputtering Method	Current (A)	Composition (at%)
I	RF	100	100	DC	0	0
II			96.5		0.02	3.5
III			41		0.05	59
IV			29.9		0.08	70.1
V			24.5		0.1	75.5
